# Non-Small Cell Lung Cancer beyond Biomarkers: The Evolving Landscape of Clinical Trial Design

**DOI:** 10.3390/jpm4030386

**Published:** 2014-06-30

**Authors:** Anastasios Dimou, Vassiliki Papadimitrakopoulou

**Affiliations:** 1 Department of Medicine, Albert Einstein Medical Center, 5501 Old York Street, Philadelphia, PA 19141, USA; E-Mail: dimouana@einstein.edu; 2 Department of Thoracic Head and Neck, The University of Texas MD Anderson Cancer Center, 1515 Holcombe Boulevard, Unit 432, Houston, TX 77030, USA

**Keywords:** targeted therapies, selumetinib, NSCLC, onartuzumab, MET, PI3K, MEK, ALK, erlotinib, afatinib, crizotinib

## Abstract

The approval of EGFR and ALK directed tyrosine kinase inhibitors materialized the concept of tailoring therapy on the basis of specific biomarkers for treating patients with NSCLC. Research for other biologics, although demonstrating clinical benefit, has been less successful so far for producing biomarkers that predict response. Blocking angiogenesis is the prototype for the agents that belong in the latter group that target specific molecules, yet they are currently approved for relatively unselected groups of patients. In order to meet the goal of personalizing care in the various settings of NSCLC, a wealth of biologics and compounds are currently being tested in clinical trials in different phases of clinical development. In a subset of the relevant studies, a biomarker perspective is appreciated. This review summarizes the clinical rationale of the major ongoing phase II and III NSCLC studies that employ targeting specific molecules with novel agents, as well as innovative strategies, and includes a comparative discussion of the different designs.

## 1. Introduction

NSCLC is the leading cause for cancer-related death and accounts for over a million deaths per year worldwide [1]. The disease is more likely to be diagnosed at a later stage, at a point when only palliative treatment is available [2]. The benefit from chemotherapy with the introduction of platinum-based doublets has reached a plateau, with most of the patients getting exposed to the side effects of chemotherapy and the minority experiencing an objective response [3]. Advances in understanding the molecular pathways of oncogene addiction have led into the development of drugs that specifically target a protein with special significance for the cancer cells. These kinds of targeted therapies carry the promise of affecting the tumor while sparing the normal tissues; the term “targeted” refers to the presence of a specific target that contributes preferentially to tumor biology. 

Lung cancers are markedly diverse [4,5]. Not only different histotypes [6], but, also, addiction to different pathways/molecules determines differential response to several compounds [7]. A targeted therapy might be improving clinical endpoints in a subgroup of patients with certain tumor histologic or molecular features, while this is not true for the rest of the patients. One additional element that has rapidly evolved is the genome theory of cancer that has been dramatically accelerated as a result of the knowledge gained by comprehensive genomic profiling of lung cancers [4,8,9] and the resulting push in using molecular genotyping in guiding the clinical care of lung adenocarcinoma patients. Taken together, the diversity of the disease with the specificity of the modern biologics raises the need for the identification of biomarkers that can serve as companion diagnostics to select the patients who will benefit from the particular drug while sparing the rest from the potential side effects. The investigation of such diagnostic tools has traditionally been grounded on *ad hoc* analysis of archived tissue, or the testing of targeted therapies in studies that enroll patients with certain biomarker profiles only. 

Certain targeted therapies have proven to improve the outcomes in unselected populations with NSCLC; bevacizumab targets VEGF and is established in the treatment of non-squamous carcinomas in addition to chemotherapy [10]. On the other hand, some of the targeted therapies are used in conjunction with certain companion diagnostics: EGFR tyrosine kinase inhibitors (TKIs) and crizotinib have been approved in the first line of NSCLC in the case of activating mutations in the tyrosine kinase domain of *EGFR* and the presence of anaplastic lymphoma kinase (*ALK*) translocations, respectively [7]. Particularly, the implementation of *EGFR* mutations in treatment decisions was incorporated in clinical practice several years after they were first described, whereas the approval of crizotinib in the *ALK* positive population was much faster. This paradigm shift in the approval of targeted therapies largely depends on the evolution of the clinical trial scheme from the traditional, conventional chemotherapy studies to genomics-driven, innovative designs of a novel series of modern trials.

Here, we review the ongoing trials in NSCLC that utilize targeted therapies, and we particularly focus on the individual designs. We aim to underscore modern strategies in clinical research with the prospective to advance the field in the least time and resource-wasting manner.

## 2. Methods

We searched the website “www.clinicaltrials.gov” with the following terms: “NSCLC” AND “targeted therapies”, “NSCLC” AND “MET”, “NSCLC” AND “ALK”, “NSCLC” AND “PARP”, “NSCLC” AND “MEK”, “NSCLC” AND “PI3K”, “NSCLC” AND “AKT”, “NSCLC” AND “HER2”, “NSCLC” AND “VEGF”, “NSCLC” AND “SRC”, “NSCLC” AND “erlotinib”, “NSCLC” AND “gefitinib”. The search was limited to phase II and phase III open studies only. We included only studies for advanced or metastatic cancer and excluded studies with curative treatments, like surgery or radiation. Finally, we selected to present 19 studies that summarize the spectrum of ongoing clinical research on targeted therapies in NSCLC. When relevant, we reviewed the individual designs and preliminary data in the PubMed and the American Society of Clinical Oncology (ASCO) virtual meeting databases.

## 3. Description and Discussion of Studies

We identified a number of studies that test the efficacy of a specific targeted therapy in a fairly unselected group of patients with NSCLC in a scheme that does not use companion diagnostics to determine the treatment arm. These studies permit for a broader exploratory design with the potential of *ad hoc* biomarker analysis. Although the experimental agents are biologics that target certain molecules, they can demonstrate efficacy in patient groups that might be missed if the population of the study is limited on the basis of a predefined test. This is particularly true with agents for whom our understanding of the biology is incomplete, in combinations of targeted therapies with chemotherapy or in creative combinations of biologics. A different set of trials have been designed on the basis of a presumed pharmacodynamics that determine the efficacy of a targeted agent in a biomarker-defined population. These studies have the potential of providing positive outcomes that would otherwise be diluted in the unselected population of patients. In addition, this type of companion diagnostic development is superior to the *ad hoc* analysis of archived tissue, as the latter is not part of the initial treatment allocation protocol and, therefore, subject to different kinds of bias. Moving forward, a third type of study design tests individual patients for the presence of an entire set of biomarkers that reflect the underlying predominant biology. In contrast to the aforementioned study types, in this last group of trials, patients are registered to more than one targeted therapies, which are paired with the multiplex biomarker analysis. Table 1 summarizes the designs of the individual studies.

**Table 1:** Comparative description of study designs.

Study Identifier Name Sponsor	Study Design	Setting	Biomarker/Population Selection	Treatment Arms	Primary Endpoint	Tissue Requirement for Biomarker Analysis
3.1. Trials with EGFR Pathway Targeted Therapies						
NCT00637910 TAILOR Fatebenefratelli and Ophthalmic Hospital	Phase III Randomized Open label	2nd line	*EGFR* WT	Erlotinib Docetaxel	OS	Archived tissue
NCT01360554 ARCHER 1009 Pfizer	Phase III Randomized Double blind Superiority	2nd line 3rd line	none	Erlotinib Dacomitinib	PFS	Archived tissue
NCT01466660 LUX-Lung 7 Boehringer Ingelheim	Phase II Randomized Open label Superiority	1st line	*EGFR* mut Adenocarcinoma	Gefitinib Afatinib	PFS	No
NCT01523587 LUX-Lung 8 Boehringer Ingelheim	Phase III Randomized Open label Superiority	2nd line	Squamous cell carcinoma	Erlotinib Afatinib	PFS	Archived tissue
NCT01487265 SCRI Development Innovations, LLC/Novartis	Phase II Single arm	2nd line 3rd line 4th line	EGFR TKI sensitive	Erlotinib plus BKM120	PFS at 3 months	Archived tissue
NCT01294306 NCI	Phase II Single arm	Any line	Erlotinib sensitive	Erlotinib plus MK2206	EGFR mut: ORR	Archived tissue
					EGFR WT: DCR	
NCT01229150 NCI	Phase II Randomized Open label	2nd line 3rd line	*KRAS* mut	*KRAS* mut: Selumetinib Selumetinib plus erlotinib	*KRAS* mut: ORR	Archived tissue
			*KRAS* WT	*KRAS* WT: Erlotinib Erlotinib plus selumetinib	*KRAS* WT: PFS	
3.2. Trials with ALK Pathway Targeted Therapies						
NCT01801111 Hoffmann-La Roche	Phase II Single arm	2nd line or higher	*ALK* translocation Prior progression on crizotinib	Erlotinib plus alectinib	ORR	No
NCT01449461 Ariad Pharmaceuticals	Phase II Single arm	Any line	*ALK* translocation Prior progression on crizotinib	AP26113	ORR	Archived tissue
			*ALK* translocation Crizotinib naive			
3.3. Trials with MET and EGFR Pathway Combination Targeted Therapies						
NCT01456325 MetLung Hoffmann-La Roche	Phase III Randomized Double blind	2nd line 3rd line	MET positive	Erlotinib Erlotinib plus onartuzumab	OS	Archived tissue
3.4. Trials with Angiogenesis and EGFR Pathway Combination Targeted Therapies						
NCT01562028 BELIEF European Thoracic Oncology Platform/Spanish Lung Cancer Group	Phase II Single arm	1st line	*EGFR* mut Non Squamous	Erlotinib plus bevacizumab	PFS	Archived tissue
NCT01532089 Academic and Community Cancer Research United	Phase II Randomized Open label	1st line	*EGFR* mut Non Squamous	Erlotinib Erlotinib plus bevacizumab	PFS	No
3.5. Trials with Targeted Therapies from Multiple Pathways						
NCT01306045 NCI	Phase II Non randomized Open label	EGFR mut: 1st line or higher	*EGFR* mut	*EGFR* mut: erlotinib *KRAS*, *NRAS*, *HRAS*, or *BRAF* mut: selumetinib	ORR	Archived tissue
		Other groups: 2nd line or higher	*KRAS*, *NRAS*, *HRAS*, or *BRAF* mut			
			*PI3K* Activation	*PI3K* activation: MK2206		
			*HER2* activation	*HER2* activation: lapatinib		
			*PDGFR* mut or amplification or *KIT* mut	*PDGFR* mut or amplification or *KIT* mut: sunitinib		
NCT01248247 BATTLE II M.D. Anderson Cancer Center	Phase II Randomized Open label	Any line	Adaptive randomization based on ongoing analysis that attests which treatment is best in the setting of specific biomarker patterns	Erlotinib Erlotinib plus MK2206 MK2206 plus selumetinib sorafenib	8 week PFS	Real time biopsy
3.6. Trials with Therapies Inhibiting Miscellaneous Targets						
NCT00787267 TOP0801 DUKE University	Phase II Single arm	2nd line or higher	None	dasatinib	Biomarker predictors of response	Real time biopsy
NCT01514864 Bristol-Myers Squibb	Phase II Single arm	Any line	*BRAF* or *DDR2* mutations	dasatinib	ORR	No
NCT01124864 Novartis Pharmaceuticals	Phase II Single arm	3rd or higher	*EGFR* mut	AUY922	Response at 18 weeks	Archived tissue Real time biopsy only for the modified EGFR mut group
			*KRAS* mut			
			*EGFR* and *KRAS* WT			
			*ALK* translocation			
			Modified *EGFR* mut (*EGFR* mut with prior response to EGFR TKI)			
NCT01788332 Lisette Nixon	Phase II Randomized Double blind	Maintenance after 1st line chemotherapy	Only patients with response to first line chemotherapy will be randomized	Olaparib Placebo	PFS	Archived tissue
NCT01560104 AbbVie	Phase II Randomized 2:1 Double blind	1st line	*EGFR* wild type	Carboplatin plus paclitaxel plus veliparib Carboplatin plus paclitaxel plus placebo	PFS	Archived tissue

Abbreviations: TKI, tyrosine kinase inhibitor; PFS, progression-free survival; OS, overall survival; mut, mutant; WT, wild-type; ORR odds ratio.

### 3.1. EGFR Pathway Targeted Therapies

Research in mutations in the tyrosine kinase domain of the epidermal growth factor receptor (EGFR) has established the use of EGFR tyrosine kinase inhibitors in the first line treatment of patients with such activating mutations [11]. Hence, the role of erlotinib and gefitinib in the *EGFR* wild-type population in the second line setting emerges as a reasonable milestone in guiding the selection of patients that would benefit the most from EGFR tyrosine kinase inhibition. Among the group of trials that address this question, the Tarceva Italian Lung Optimization Trial (TAILOR) study (NCT00637910) is directly comparing erlotinib with docetaxel in patients with wild-type *EGFR* who have progressed after first line chemotherapy [12,13]. The TAILOR investigators randomized the patients with a 1:1 minimization protocol that offers the advantage of reducing the imbalance in treatment group allocation in various subpopulations. The study was designed as a superiority trial with the power to prove a 33% better overall survival in the docetaxel arm. Interestingly, the design does not allow cross over from one arm to the other upon disease progression, which makes overall survival more relevant as the primary endpoint that reflects the effect of the study intervention. Instead, this requirement will withhold docetaxel from patients who progress on erlotinib and would otherwise be eligible for the drug. Results from the TAILOR study showed that the erlotinib arm had worse progression-free survival compared to the docetaxel arm irrespective of the *KRAS* status. This is in contradiction to the data from the INTEREST study [14], which nonetheless compared gefitinib with docetaxel regardless of *EGFR* mutation information and to the data from the Tarceva In Treatment of Advanced NSCLC (TITAN) study [15], which compared erlotinib with chemotherapy in *EGFR* wild-type patients. The latter study was designed as a superiority rather than non-inferiority study, and the lack of difference between the arms does not necessarily translate into equivalence. 

The first generation of tyrosine kinase inhibitors is EGFR specific and causes reversible inhibition of the receptor. Dacomitinib and afatinib belong to a new group of molecules that irreversibly inhibit several members of the HER family. The former is an oral irreversible pan HER inhibitor that was shown to achieve a superior progression-free survival compared to erlotinib in a phase II randomized study [16], especially in *KRAS* wild-type tumors and regardless of the *EGFR* mutational status. There are currently three studies to compare directly the irreversible inhibitors with erlotinib or gefitinib (NCT01360554, NCT01466660 and NCT01523587); the individual characteristics for every study are described in Table 1. Progression-free survival is the primary outcome in these studies. In the first of these studies (NCT01360554), erlotinib is compared to dacomitinib in a phase III double blind study in patients who have progressed after first line treatment. Patients are enrolled regardless of *EGFR* mutations. The second trial (NCT01466660) is a phase II open label study comparing gefitinib with afatinib as the front line therapy for patients with lung adenocarcinoma with *EGFR* mutations. Last, but not least, the third trial (NCT01523587) is a phase III open label study comparing erlotinib with afatinib in a second line of patients with squamous cell carcinoma of the lung. The current approval for the tyrosine kinase inhibitors portends the clinical relevance of the comparison between reversible and irreversible inhibition of EGFR in the first line treatment of the patients who bear mutations, in patients with acquired resistance to erlotinib or gefitinib and in pretreated patients with wild-type *EGFR* who are considered candidates for EGFR inhibition in the second or third line. The failure of inhibiting transmembrane receptors to translate into meaningful clinical benefit for the patient has often been attributed to the constant and independent activation of downstream molecules. In this context, the idea of blocking targets at various points in the PI3K/AKT/mTOR and RAS/RAF/MEK molecular cascades that drive the tumors has evolved into a promising concept of personalizing care in NSCLC. BKM120 is a putative PI3K inhibitor that is tested in combination with erlotinib in patients who were initially sensitive to erlotinib monotherapy and have developed secondary resistance at the time of enrollment to the study (NCT01487265). In a parallel study (NCT01294306), the AKT inhibitor MK-2206 is combined with erlotinib again to treat patients with prior sensitivity to TKI therapy. Both studies include a phase II protocol that does not include a comparator arm, and while the former will look at the progression free survival at three months, the latter will focus on the objective response and disease control rate as indicated by the response and stable disease rates combined. Prior sensitivity to erlotinib or tyrosine kinase inhibitors is defined as an objective response or disease control. Relevant to these trials, the classic AKT inhibitor enzastaurin failed to add benefit to conventional chemotherapy or erlotinib in unselected groups of patients with NSCLC [17,18,19]. The strategy that is proposed with the dual EGFR and PI3K or AKT inhibition in the selected population that has become refractory to erlotinib after initial response will add to the knowledge generated in the enzastaurin trials. 

The significance of specific inhibition of the RAS/RAF/MEK cascade is illustrated by the frequent presence of activating mutations at several points of the pathway. The Biomarker-integrated Approaches of Targeted Therapy for Lung Cancer Elimination 1 (BATTLE1) study depicted sorafenib as a successful drug in patients with *KRAS* mutated tumors [20], an effect that was largely attributed to RAF inhibition. Selumetinib is a promising MEK inhibitor that is currently under investigation in NSCLC. In a phase II double blind study, selumetinib plus docetaxel was compared to docetaxel plus placebo in patients with *KRAS* mutant NSCLC that had already been treated with first line platinum-based chemotherapy [21]. The study showed that the combination resulted in a non-significant improvement in overall survival and a significant improvement in progression-free survival. Interestingly, there was a striking difference in the response rate in favor of combination therapy. In fact, none of the patients who were treated with docetaxel alone had an objective response, providing further proof for the lack of efficacy of chemotherapy in the presence of *KRAS* mutations. A separate ongoing open label phase II study (NCT01229150) that uses selumetinib in the second line setting is randomizing patients after testing for the presence of *KRAS* mutations; those with *KRAS* wild-type NSCLC will be randomized to receive erlotinib *vs.* erlotinib plus selumetinib, whereas the patients with *KRAS* mutations are randomized into a selumetinib *vs.* a selumetinib plus erlotinib group. The primary endpoint is progression-free survival. MEK and EGFR inhibitors act in a synergistic mode [22] that renders the design of this trial the potential to boost the effect of EGFR inhibition. 

### 3.2. Inhibition of the ALK Pathway

The development of crizotinib as a putative ALK inhibitor and the successful treatment of *ALK* positive tumors with crizotinib [23], not only lead to the initiation of a series of studies in this particular subset of patients with NSCLC, but also created a paradigm shift in the clinical trials with targeted agents. Crizotinib was approved on the basis of a phase I study that was able to show a high objective response rate validating the convincing biologic background. The superiority of crizotinib over chemotherapy for patients with *ALK* translocations was confirmed in a phase III study [24]. Current trials in the *ALK* positive patients focus on second generation ALK inhibitors after the failure of crizotinib. Ceretinib is an ALK inhibitor that has already been announced to be highly efficient in the crizotinib-resistant ALK positive patients [25]. Two other compounds, alectinib and AP26113, are investigated in patients with *ALK* translocations who have failed treatment with crizotinib (NCT01801111 and NCT01449461, respectively). Interestingly, these are studies that look at the objective response rate as the primary efficacy endpoint.

### 3.3. Combination of MET and EGFR Pathway Inhibition

The MET receptor has been identified as an attractive target in NSCLC, because it mediates secondary resistance to EGFR TKIs [26], while gene amplification and overexpression of *MET* is present even in treatment-naive tumors and predicts worse prognosis [27]. A series of trials have been designed to investigate MET inhibition in NSCLC, and they are summarized in Table 1. In the phase III MetLUNG study (NCT01456325), patients who have been treated with one, but no more than two prior treatment lines are randomized to receive erlotinib plus onartuzumab *vs.* erlotinib plus placebo. Both patients and physicians are blinded, and the study will test overall survival as the primary outcome. All of the participants are required to have tissue available for translational analysis, while the positivity of the tumors for MET is mandatory. The assay that is employed to test for MET positivity was determined to be immunohistochemistry with SP44, which is a validated rabbit monoclonal antibody [28]. Preliminary results from the MetLUNG study did not show any improvement in overall or progression-free survival [29]. Likewise to onartuzumab, tivantinib is a MET inhibitor; the latter is a small molecule that binds to c-MET and blocks its auto activation. Despite the promising results from a phase II study that showed that the combination of tivantinib with erlotinib is superior to erlotinib alone [30], the subsequent phase III MET Inhibitor Tivantinib (ARQ 197) Plus. Erlotinib *vs* Erlotinib Plus Placebo in NSCLC (MARQEE) study failed to meet its primary endpoint of a 33% decrease in overall survival in the interim analysis and was prematurely closed. Nevertheless, these results refer to the unselected non-squamous population, and therefore, the analysis of the molecularly-defined subgroups is much expected and potentially hypothesis generating. 

### 3.4. Anti-Angiogenesis Targeted Therapies Combined with EGFR Inhibition

In recent years, anti-angiogenesis treatments have been established in combination with chemotherapy in the first line setting of patients with NSCLC and non-squamous histology [10]. In the light of data of VEGF-mediated resistance to EGFR targeting therapies [31] and the potential benefit from the dual VEGFR and EGFR inhibitor, vandetanib [32,33], new studies try to explore the effect of combining VEGF and EGFR inhibitors. The Bevacizumab and ErLotinib in EGFR Mut positive NSCLC (BELIEF) trial (NCT01562028) is testing the combination of erlotinib plus bevacizumab in treatment-naive patients with *EGFR* mutations, whereas in a second study, patients with *EGFR* mutations are randomized to receive erlotinib *vs.* erlotinib plus bevacizumab (NCT01532089). Both phase II studies will have progression-free survival as the primary endpoint in treatment naive populations. In the former study, there is a requirement for central tissue processing. Both focus on the two predominant *EGFR* mutations, the deletion in exon 19 and the L858R point mutation, and they will look at the T790M point mutation as a potential predictor of response to combination treatment. The T790M mutation is known to mediate secondary resistance to EGFR TKIs [34], yet there is evidence that it can be detected even before treatment with erlotinib or gefitinib when a high sensitivity method of detection is applied [35].

### 3.5. Trials with Multiple Targets

The principle of designing a trial that introduces a multiple targeted therapy protocol in conjunction with a molecular profiling platform is conceptualized in two phase II studies. The first is performed by the National Cancer Institute, and the second is the BATTLE2 study. In the NCI trial (NCT01306045), patients with NSCLC, SCLC or thymic malignancies who are not considered to have curable disease with surgery or radiation are assigned to one of five treatment arms with a deterministic protocol that depends on testing for a series of molecular changes: those with *EGFR* mutations will receive erlotinib, those with *KRAS*, *NRAS*, *HRAS* or *BRAF* mutations will receive selumetinib, those with *HER2* mutations or *HER2* gene amplification will receive lapatinib, those with *PI3K*, *AKT* or *PTEN* mutations will receive MK-2206, and finally, those with *KIT* or *PDGFRA* mutations will receive the multi-target agent sunitinib. The primary endpoint is the objective response rate, as well as generation of safety data. While this study includes many of the important genetic alterations that have been described in NSCLC, it carries a reasonable hypothesis about the efficacy of the different targeted agents, which integrates preclinical and pharmacodynamic data. However, it allows for the utilization of archived tissue for genetic testing rather than real-time biopsies. Patients, who have received additional treatments in the gap time period between the biopsy and enrollment in the study might have dissimilar culprit genotypes compared to what is assumed on the basis of the testing that was done as part of the study protocol.

The second of the studies that test more than one targeted therapies is the BATTLE2 trial (NCT01248247), which was designed as a phase II after the completion of the successful BATTLE1 study. The BATTLE2, likewise to the BATTLE1, is innovative for a number for reasons. First, in a similar fashion to the NCI study, it has an umbrella structure where a number of targeted agents are exploited in a way that reflects the complexity and diversity of the underlying molecular biology. Second, all patients get real-time biopsies at the time of enrollment. Third and unlike the NCI trial, patients are randomized to one of four open label arms: erlotinib, erlotinib plus MK-2206, MK-2206 plus AZD6244 and sorafenib. The randomization protocol utilizes the Bayesian principle in order to adapt the weight of each arm in the presence of a specific genotype taking into account the outcomes of patients that have already been enrolled in the trial. The adaptation of treatment allocation allows for assigning personalized treatments to patients that are most likely to benefit them; while far from carrying *a priori* assumptions about which biomarker is more likely to predict response to a targeted agent, it finally develops a heuristic biomarker-based protocol that is built within the trial. Fourth, the primary endpoint of eight-week progression-free survival allows for a more resource- and time-consuming design, while it has proven to correlate with more traditional endpoints, like the progression-free and overall survival [36]. 

### 3.6. Miscellaneous

Several other targeted therapies are tested in clinical trials in NSCLC. In particular, the inhibition of SRC as a monotherapy with dasatinib in patients with NSCLC that have failed prior regimens is tested in a phase II study (NCT00787267), whereas there is a separate dasatinib trial (NCT01514864). A unique principle is providing a rationale for the studies that target the heat shock protein 90 (HSP90): HSP90 protects numerous molecules, which are called client proteins, that the tumor might be addicted to from ubiquitin-mediated degradation. Thus, blocking hsp90 can potentially and indirectly lead to tumor cell death by deprivation of its molecular machinery [37]. A phase II single arm study in the third line or higher setting uses AUY922, which is a specific hsp90 inhibitor (NCT01124864); interestingly, it is designed to look at differences in efficacy in five different strata: *KRAS* mutant, *EGFR* mutant, *KRAS* and *EGFR* wild-type, *ALK* positive and, finally, the *EGFR* mutant that has responded to prior TKI therapy. A different strategy is suggested with compounds that inhibit the poly ADP ribose polymerase (PARP) and cause cell death when a second “hit” that affects the integrity of DNA is present, since PARP is an enzyme that is involved in the repair of DNA. The second hit might be a genetic alteration that is tumor specific or the effect of chemotherapy, like an alkylating agent. There are currently two ongoing phase II, placebo-controlled studies that use this concept of “synthetic lethality” in NSCLC (NCT01788332, NCT01560104); the former uses olaparib as a maintenance therapy in patients who have responded to standard treatment, and the latter uses veliparib in combination with chemotherapy in treatment-naive patients. 

## 4. Concluding Remarks

As the paradigm of genomic-driven clinical trial approaches is evolving, the need to address rare molecular subtypes within a reasonable timeframe has led to the recognition that large screening networks for patient recruitment and collaboration between multiple centers is needed. One such network is the Lung Cancer Mutation Consortium that aimed at genotyping 1,000 lung adenocarcinomas for a panel of well-known mutations and gene fusions and concurrently offered participation to a number of targeted therapy multicenter clinical trial addressing these molecular subtypes [38].

A second project that is currently being launched is a phase II/III biomarker-driven master protocol refractory to frontline therapy squamous cell lung cancer. Similar to the molecular landscape in lung adenocarcinoma, progress in genotyping is being made for squamous cell carcinoma of the lung (SCCA) primarily led by TCGA, but also other sequencing efforts [39,40]. A number of the alterations that have been identified are targetable, but relatively uncommon, while others are more common, but not as readily actionable. Lung SCCA remains an “orphan” group, where substantial developments in therapeutics have yet to be seen and all of the targeted therapies so far approved in NSCLC are largely ineffective. This approach will provide the basis for FDA approval of new drugs with matching companion diagnostics. If this multi-arm, master protocol strategy is successful, this type of biomarker-driven umbrella protocol could be used for registration trials in other settings.

Finally, immunotherapies have introduced a different concept in personalized care in lung cancer by training the immune system to recognize tumor-associated antigens (TAAs) that are specifically expressed in cancer cells. This has been attempted with the aid of vaccines that have generated a shift in our understanding of NSCLC from a poorly to a highly immunogenic tumor [41]. A number of different vaccine design approaches range from protein or peptide-based to tumor cell lysates and dendritic cell-based vaccines [42]. It has been postulated that advanced tumors have already developed the means to escape immunosurveillance [43]. Therefore, the stimulation of a cancer-specific immune response needs to be derived from the manipulation of the tumor’s ability to interact with the immune system. The most exciting development in that respect is the development of novel antibodies targeting immune regulatory checkpoints for cancer therapy. Immune checkpoints are the inhibitory pathways that are vital for maintaining self-tolerance and modulating the duration and amplitude of immune responses in peripheral tissues [44]. Ipilimumab, a mAb against the immune checkpoint marker, CTLA-4, a regulatory molecule on the surface of activated T-cells involved in the maintenance of T-cell homeostasis. The programmed death 1 (PD-1) receptor expressed by activated T-cells is another key immune checkpoint receptor with a negative regulatory role when engaged by its ligands, PDL1 (also known as B7-H1) and PD-L2 (also known as B7-DC), within the tumor microenvironment [45]. Two recent phase I trials have investigated the safety and efficacy of mAb against PD-1 and PD-L1. Topalian *et al.* [46] conducted a phase I study examining the safety and efficacy of a fully humanized mAb, directed at PD-1 blockade and showing a cumulative objective response rate of 18% in NSCLC patients, while the second study utilized PD-L1-specific mAb [47] that inhibits PD-1–PD-L1 binding, with 10% objective response in NSCLC. These studies have confirmed PD-1 and PD-L1 as very promising targets for future NSCLC trials and lend themselves to combination approaches, not only between immunotherapeutics, but also with targeted therapeutics within distinct molecular subsets [48].

An exciting new series of clinical trials that investigate the role of novel targeted therapies aim to advance the available therapeutic options and achieve the endpoint of personalized care in NSCLC. A series of innovations in the recent clinical research design can expedite the approval of more efficient drugs and the development of accurate companion diagnostics to guide treatment decisions. The success of ALK inhibitors on the basis of impressive objective response rates in phase I studies is indicative of the value of surrogate clinical endpoints that correlate very well with more traditional and resource consuming study objectives, like overall survival. On the other hand, the adaptation randomization protocol that is used in the BATTLE study series offers the ability to develop powerful biomarkers without any pre-analytical assumptions on the performance of the different targeted agents. Finally, studies with multi-armed registration, on the one hand, reflect the diversity of patients with NSCLC and, on the other hand, support the development of master protocols, where novel targeted agents can be added to a basic “standard of care” trial design. The latter not only augments consistency in comparisons between different agents, but also accelerates the approval of efficient drugs. 

Novelties in the design of clinical trials in NSCLC offer certain advantages in disentangling the role of modern drugs in the era of targeted therapies. The implementation of such tools will optimize the use of resources and will improve the prognosis of patients with lung cancer.
